# Effects of Arbuscular Mycorrhizal Fungi and Biochar on Growth, Nutrient Absorption, and Physiological Properties of Maize (*Zea mays* L.)

**DOI:** 10.3390/jof8121275

**Published:** 2022-12-05

**Authors:** Jiahua Sun, Qiong Jia, Yi Li, Ting Zhang, Jiayuan Chen, Yanan Ren, Kanglong Dong, Shuai Xu, Nan-Nan Shi, Shenglei Fu

**Affiliations:** 1 College of Geography and Environmental Science, Henan University, Kaifeng 475001, China; 2 Key Laboratory of Geospatial Technology for the Middle and Lower Yellow River Regions, Henan University, Ministry of Education, Kaifeng 475001, China; 3 Dabieshan National Observation and Research Field Station of Forest Ecosystem, Henan University, Kaifeng 475001, China

**Keywords:** AMF, biochar addition, interactive effect, nutrient uptake, physiological properties

## Abstract

Arbuscular mycorrhizal fungi (AMFs) and biochar are two common alternatives to chemical fertilizers applied to soil to improve crop growth. However, their interactive effects on maize (*Zea mays* L.) growth, nutrient absorption, and physiological properties remain poorly understood. In this study, maize plants were grown in pots treated with biochar and AMFs *Diversispora eburnea*, alone or in combination. The results showed that the individual application of AMFs or biochar increased maize growth and mineral contents in shoots and roots (including P, K, Ca, Na, Mg, Fe, Mn, and Zn). The chlorophyll a, chlorophyll b, and total chlorophyll contents in AMF-treated leaves were significantly higher than those in the control treatment group. However, AMFs had no synergistic effects with biochar on maize growth, nutrient absorption, nor photosynthetic pigments. The application of biochar to the soil significantly reduced mycorrhizal colonization by 40.58% in the root tissues, accompanied by a significant decline in mycorrhizal dependency from 80.57% to −28.67%. We conclude that the application of biochar and AMFs can affect maize growth, nutrient uptake, and physiological properties. Our study can provide vital information for further resource use optimization in agroecosystems.

## 1. Introduction

Given the limited cultivated land area (38% of the global land surface), escalating global population pressure, and extreme weather, the sufficiency of the global food supply to feed the human population has been challenged [1]. To address this challenge, excessive chemical fertilizers have been applied to fields to increase crop yields [2]. According to data from China Statistical Bureau, China is the largest consumer of agricultural chemicals in the world, with more than 30% of global fertilizers being applied to only 9% of the world’s cropland. However, the excessive use of chemical fertilizers has resulted in considerable adverse environmental impacts, such as land and water eutrophication, greenhouse gas emissions, and biodiversity loss [3,4]. Therefore, it is critical to develop alternatives to chemical fertilizers to improve crop productivity without threatening soil health and agricultural ecosystems.

In recent years, soil microorganisms have been presented as safe and effective biofertilizers [5]. Arbuscular mycorrhizal fungi (AMFs) are essential components of the rhizosphere microflora in natural environments, accounting for 20–30% of total soil microbial biomass and forming obligatory mutualism with 80% of vascular plants [6]. In addition to increasing the absorptive surface area of their host plant root systems, AMFs can promote nutrient uptake by transferring nutrients (particularly P) from the soil to the crop via their external mycelium [7]. AMFs can also promote soil photosynthetic activity, improve the water–plant relationship, and cause biochemical and physiological changes in plants using a variety of compounds and molecules, such as auxins, cytokinins, gibberellins, and bioactive organic compounds [8,9].

Biochar is a carbon-rich substance that is produced via the pyrolysis of different organic materials in an oxygen-limited environment. It has a unique porous structure with great biochemical stability, as well as a high adsorption capacity for sorbing and releasing mineral nutrients owing to its large specific surface area [10]. When added to soil, biochar can improve soil organic carbon (SOC) content, cation exchange capacity (CEC), soil porosity, and the bioavailability of minerals such as K, Ca, Mg, and P, resulting in improved root development and nutrient uptake [11,12,13]. Biochar may also affect soil nutrients by altering microbial and fungal metabolism as well as diversity, both of which can influence the availability and quality of soil nutrients [14]. Moreover, the majority of C in biochar persists in the soil for hundreds to thousands of years, making biochar attractive as a soil amendment option to replace mineral fertilizers [15].

Previous studies have shown that soil amendment using biochar can promote the abundance and infection rate of AMFs [16,17] by modifying soil properties and altering microbial activity [16,17,18]. Biochar coupled with AMFs has been demonstrated to boost plant growth, decrease disease severity, and increase productivity [19]. Under soil water deficit conditions, biochar application can increase soil water-holding capacity and promote the growth of AMFs [20]. Using a combination of biochar and AMFs can result in an increase in total root length and root development in strawberry crops [21]. Despite that, knowledge on the combined effect of AMFs and biochar on plant growth, nutrient absorption, and physiological properties is still deficient [22]. Therefore, testing the combination of biochar and AMFs could provide vital information for further resource use optimization in agroecosystems. 

Maize (*Zea mays* L.) is one of the most important cereal crops in agricultural ecosystems, both in terms of cultivated area and amount produced worldwide. The demand for maize is increasing and will likely continue to increase due to the industrial use of maize by-products, biofuels, and food supply for the growing human population [23]. In the present study, maize seedlings grown in soil were inoculated with AMFs or biochar to evaluate whether the use of biochar and AMFs, alone or in combination, improved nutrient uptake, physiological properties, and maize growth. Based on these objectives, we hypothesized that (1) the individual application of AMFs or biochar promotes maize growth, nutrient uptake, and physiological properties compared with the control and (2) the combined application of AMF and biochar promotes maize performance more than either treatment alone.

## 2. Materials and Methods

### 2.1. Materials

Topsoil (0–20 cm) was collected from an experimental field at Henan University, Henan Province, China (34°49′3″ N, 114°18′38″ E). To remove plant residue and stones, the soil was homogenized and sieved through a 2 mm mesh and was then autoclaved at 120 °C for 2 h. The soil (pH 8.32) contained 13.6 g kg^−1^ of organic carbon, 1.1 g kg^−1^ of total N, 0.50 mg kg^−1^ of total P, 2.75 mg kg^−1^ of NH_4_^+^-N, 45.39 mg kg^−1^ of NO_3_^−^-N, and 3.7 mg kg^−1^ of available P. 

The AMF species, *Diversispora eburnea* BGC HK02C, used in the present study was provided by Beijing Academy of Agriculture and Forestry, Beijing, China. The AMF species was identified based on morphological characteristics and 18S rDNA sequence analysis, and the sequences were deposited in GenBank (accession number KT152858). 

The inocula were propagated on maize (*Zea mays* L.) in autoclaved soil/vermiculite (1:1, *v*/*v*) substrate for six months. The inoculum was composed of spores (50 spores per gram of soil), hyphae, root pieces, and soil. 

The biochar used in this study was prepared using maize straw. Cleaned and air-dried maize straw was cut into small segments (~10 cm in length), fed into a biochar reactor with a tight-fitting lid, and pyrolyzed at 550 °C for 4 h in a muffle furnace. The biochar was then dried, crushed, and passed through a 2 mm mesh. The biochar had a total C content of 597.7 g kg^−1^, total N content of 13.4 g kg^−1^, total P content of 2.47 g kg^−1^, and CEC of 17.0 cmol kg^−1^.

### 2.2. Experimental Setup

The experiment was set up using a completely randomized factorial design. Four amendment treatments were included in this experiment: (1) CK (control, without application of AMFs or biochar); (2) A (individual application of AMFs); (3) B (individual application of biochar); and (4) AB (combined application of AMFs and biochar). Each treatment had 5 replicates, yielding 20 experimental pots. Biochar was applied to the soil at a rate of 3% (*w*/*w*) in the biochar treatments. Each pot was inoculated with 50 g of the inoculum for mycorrhizal treatments or 50 g of the autoclaved inoculum for non-mycorrhizal treatments. A total of 10 mL of microbial filtrate (10 μm pore size) was applied to provide similar microflora. Maize seeds (variety: Zhengdan 958) were surface-sterilized with 0.5% NaClO solution (*v*/*v*) for 5 min, rinsed in distilled water, and then germinated on wet filter paper in a sterile culture dish at 25 °C. Four pre-germinated seeds were sown in each pot (30 cm in height and 13.5 cm in diameter) containing 2 kg of soil. Maize seedlings were thinned to two uniform seedlings per pot after emergence.

The experiment was conducted from 14 May to 14 July 2021. Maize seedlings were watered to 65% of their water-holding capacity daily. The greenhouse temperature was in the range of 15–28 °C during the experimental period. No additional nutrients were used in the experiments. The positions of all trial pots were randomized every two weeks. Maize height, basal diameter, and leaf number were measured every two weeks.

### 2.3. Harvest and Analysis

During harvest, maize shoots were cut at the soil surface, and roots were carefully separated from the soil. The roots were divided into two subsamples, and the fresh mass was determined for both. The shoots and one root subsample were oven-dried at 80 °C to a constant weight and then weighed. The other root subsample was used for AMF colonization observation, and its dry mass was calculated by multiplying the fresh mass by the dry-to-fresh mass ratio of the oven-dried root subsamples. Samples (0.5 g) of finely ground tissues (roots and shoots) were wet-digested using a mixture of concentrated HNO_3_ and HClO_4_ (4:1 *v*/*v*, guaranteed reagent), and the digests were adjusted to a final volume of 50 mL using deionized water. The P, K, Ca, Na, Mg, Fe, Mn, and Zn contents in shoots and roots were determined using inductively coupled plasma-atomic emission spectrometry (ICP-AES, Thermo Fisher Scientific, Waltham, England). AMF colonization was quantified under a microscope (×200) using the line intersect method after the roots had been cleared in 10% KOH solution (*w*/*v*) and stained with acid fuchsin [24]. Soil pH was measured using a pH meter in a 1:2.5 (*w*/*v*) suspension of soil and deionized water. Soil available N (NH_4_^+^-N and NO_3_^−^-N) was determined using SmartChem 200 automatic chemical analyzer (AMS Group Westco, Rome, Italy) and measured using dual wavelength spectrophotometry and the indophenol blue method. Available P (A-P) was extracted using 0.5 M NaHCO_3_ at a pH of 8.5 and quantified using SmartChem 200 automatic chemical analyzer. Soil dissolved organic carbon (DOC) was determined using multi N/C 2100/2100S TOC Analyzer (Element, Jena, Germany). Soil microbial biomass carbon (MBC) and microbial biomass nitrogen (MBN) were determined using the chloroform fumigation method and quantified using multi N/C 2100/2100S TOC Analyzer (Element, Jena, Germany). The soil alkaline phosphatase activity, and chlorophyll and carotenoid contents in the leaves were determined using BC0285, BC0995, and BC4335 assay kits, respectively (Solarbio, Beijing, China). The enzyme activity unit of alkaline phosphatase was defined as 1 nanomole of phenol released per gram of soil per day at 37 °C (nmol·g^−1^·d^−1^).

### 2.4. Data Analysis

SPSS software (version 25.0) was used for the data analysis. A three-way ANOVA was applied to examine the effects of AMFs, biochar, sampling time, and their interactions on plant height, basal diameter, and leaf number of maize. A two-way ANOVA was applied to examine the effects of AMFs, biochar application, and their interaction on maize shoot, root, and total biomass; root–shoot ratio; photosynthetic pigments; nutrient uptake; soil chemical properties; and alkaline phosphatase activity. A one-way ANOVA followed by a Duncan’s test was used to evaluate significant differences among different treatments at *p* < 0.05. The mycorrhizal dependency (MD) presents the degree to which a plant relies upon the mycorrhizal condition to produce its maximum growth at a given level of soil fertility. It is calculated using the following formula:

$$\mathrm{MD}\%=\left({\mathrm{TB}}_{\mathrm{AMF}}-{\mathrm{TB}}_{\mathrm{NM}}\right)/{\mathrm{TB}}_{\mathrm{NM}}\times 100\%$$

where TB_AMF_ and TB_NM_ represent the total biomass of AMF-inoculated maize and non-inoculated maize, respectively.

## 3. Results

### 3.1. Mycorrhizal Colonization Rate

AMF colonization was not observed in the roots of non-inoculated maize at harvest. Inoculated maize had a mycorrhizal colonization rate ranging from 31.52% to 52.77%. The application of biochar decreased the mycorrhizal colonization rate significantly, by 40.58%, relative to that of the AMF-only treatment (Table 1). The mycorrhizal colonization rate was significantly negatively correlated with soil available P and positively correlated with chlorophyll a content, according to the Pearson correlation analysis (Figure S1).

### 3.2. Soil Chemical Characteristics and Phosphatase Activity

The two-way ANOVA showed that soil NO_3_^−^-N, available P, DOC, and MBC were significantly influenced by AMFs (Table 2). Soil NH_4_^+^-N, NO_3_^−^-N, available P, DOC, MBC, and MBN were significantly influenced by biochar (Table 2). Soil NH_4_^+^-N, NO_3_^−^-N, available P, MBC, and alkaline phosphatase activity were significantly influenced by the interaction between AMFs and biochar (Table 2). 

Soil NH_4_^+^-N, NO_3_^−^-N, and MBC were significantly decreased by both individual and dual applications of AMFs and biochar compared with the control treatment (Table 3). Furthermore, MBN was significantly decreased by 33.3% in the biochar treatment group compared with the control treatment group (Table 3). In contrast, the dual application of AMFs and biochar significantly raised soil DOC by 49.9%, while biochar alone and the combination of AMFs and biochar increased available P by factors of 11.1 and 8.1, respectively (Table 3). Soil alkaline phosphatase activity was significantly increased by AMF and biochar application by factors of 4.93 and 5.02, respectively (Table 3).

### 3.3. Maize Growth and Mycorrhizal Dependency

**Figure 1:**
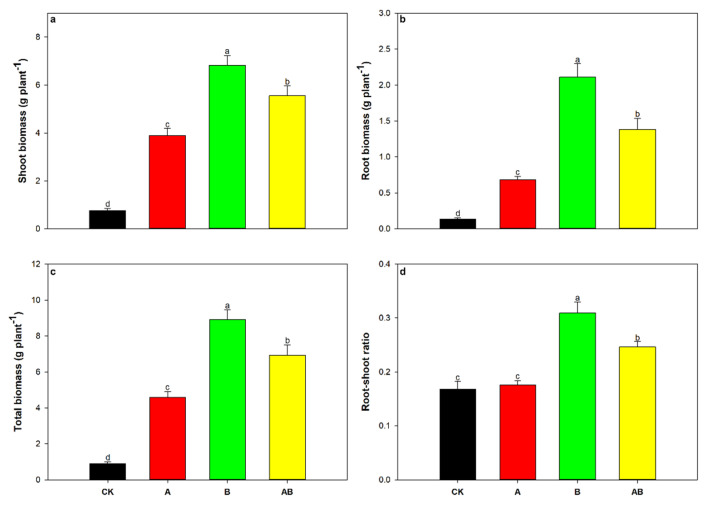
Effects of arbuscular mycorrhizal fungi and biochar on maize (**a**) shoot biomass, (**b**) root biomass, (**c**) total biomass, and (**d**) root–shoot ratio. Data are means of five replicates ±SEs. Different letters above the columns indicate significant differences according to Duncan’s test at *p* < 0.05. CK, control; A, arbuscular mycorrhizal fungi; B, biochar; AB, arbuscular mycorrhizal fungi and biochar.

The two-way ANOVA showed that maize shoot biomass, root biomass, total biomass, and root–shoot ratio were significantly influenced by biochar, as well as by the interaction between biochar and AMFs (Table 2, Figure 1a–d). The shoot and total biomass were also significantly influenced by AMFs alone (Table 2, Figure 1a,c). Compared with the control treatment, individual or dual application of AMFs and biochar significantly increased the shoot biomass of maize by factors of 4.2, 8.0, and 6.3, respectively (Figure 1a). Shoot biomass was the greatest when biochar was applied, followed by the combination of AMFs and biochar, AMFs alone, and the control treatment (Figure 1a). A similar trend was also observed in the root and total biomass of maize (Figure 1b,c). Compared with the control treatment, the root–shoot ratio was significantly increased by the application of biochar alone and the dual application by 82.4% and 47.1%, respectively (Figure 1d). Moreover, the mycorrhizal dependency of maize was 80.57% when biochar was absent, but it decreased to −28.67% after biochar addition (Table 4).

**Figure 2:**
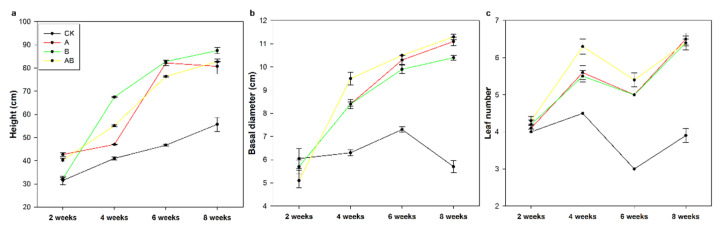
Effects of arbuscular mycorrhizal fungi and biochar on (**a**) maize height, (**b**) basal diameter, and (**c**) leaf number. Data are means of five replicates ±SEs. CK, control; A, arbuscular mycorrhizal fungi; B, biochar; AB, arbuscular mycorrhizal fungi and biochar.

The three-way ANOVA showed that the plant height, basal diameter, and leaf number of maize were significantly influenced by AMFs, biochar, sampling time, and their interactions (Table 5). Maize height was significantly improved by individual and combined applications of AMFs and biochar for the entire growth period, but biochar had no significant effects on maize height during the first two weeks (Figure 2a). Maize basal diameter and leaf number were significantly increased by individual and dual applications of AMFs and biochar after the fourth week (Figure 2b,c). In addition, there were no significant differences in maize height nor leaf number among the different application treatments at harvest (Figure 2a,c). However, the basal diameter of maize was significantly lower in the biochar treatment group than in the AMF application group or the combined treatment group at harvest (Figure 2b).

### 3.4. Photosynthetic Pigments

**Figure 3:**
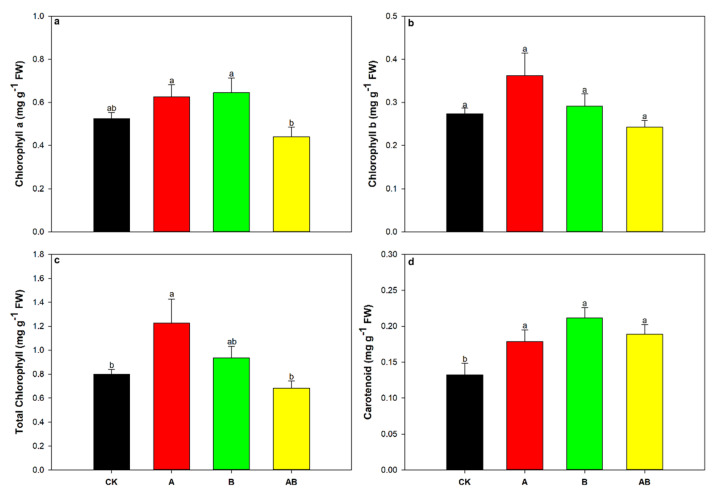
Effects of AMFs and biochar on (**a**) chlorophyll a, (**b**) chlorophyll b, (**c**) total chlorophyll, and (**d**) carotenoid contents. Data are means of five replicates ±SEs. Different letters above the columns indicate significant differences according to Duncan’s test at *p* < 0.05. FW, fresh weight; CK, control; A, arbuscular mycorrhizal fungi; B, biochar; AB, arbuscular mycorrhizal fungi and biochar.

Chlorophyll a, chlorophyll b, total chlorophyll, and carotenoid content in maize leaves were significantly influenced by the interaction between AMFs and biochar (Table 2). The carotenoid content was also significantly influenced by biochar alone (Table 2). The chlorophyll a, chlorophyll b, and total chlorophyll contents were significantly enhanced by the individual application of AMFs compared with the control (Figure 3a–c). However, the individual application of biochar had no significant effects on the chlorophyll a, chlorophyll b, nor total chlorophyll content (Figure 3a–c). There was a slight increase in the carotenoid content in the individual and dual application treatments, but the effect was not statistically significant (Figure 3d).

### 3.5. Mazie Nutrient Uptake

**Figure 4:**
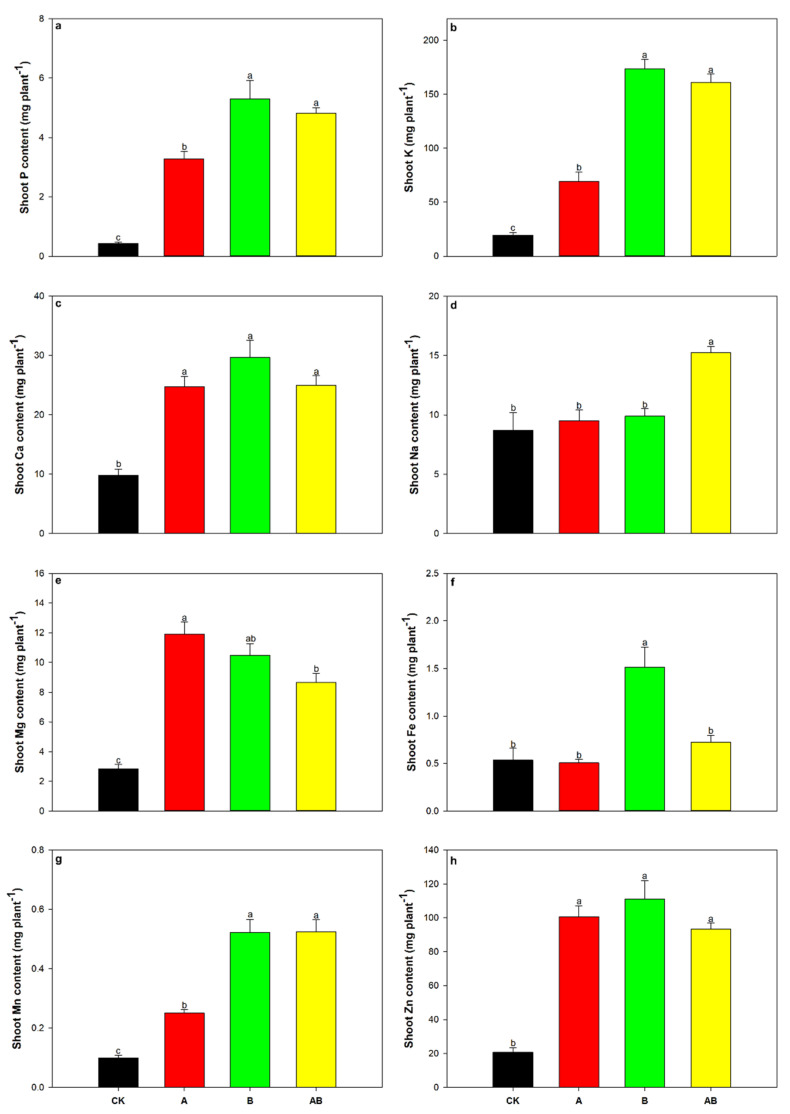
Effects of AMFs and biochar on shoot (**a**) P, (**b**) K, (**c**) Ca, (**d**) Na, (**e**) Mg, (**f**) Fe, (**g**) Mn, and (**h**) Zn contents. Data are means of five replicates ±SEs. Different letters above the columns indicate significant differences according to Duncan’s test at *p* < 0.05. CK, control; A, arbuscular mycorrhizal fungi; B, biochar; AB, arbuscular mycorrhizal fungi and biochar.

**Figure 5:**
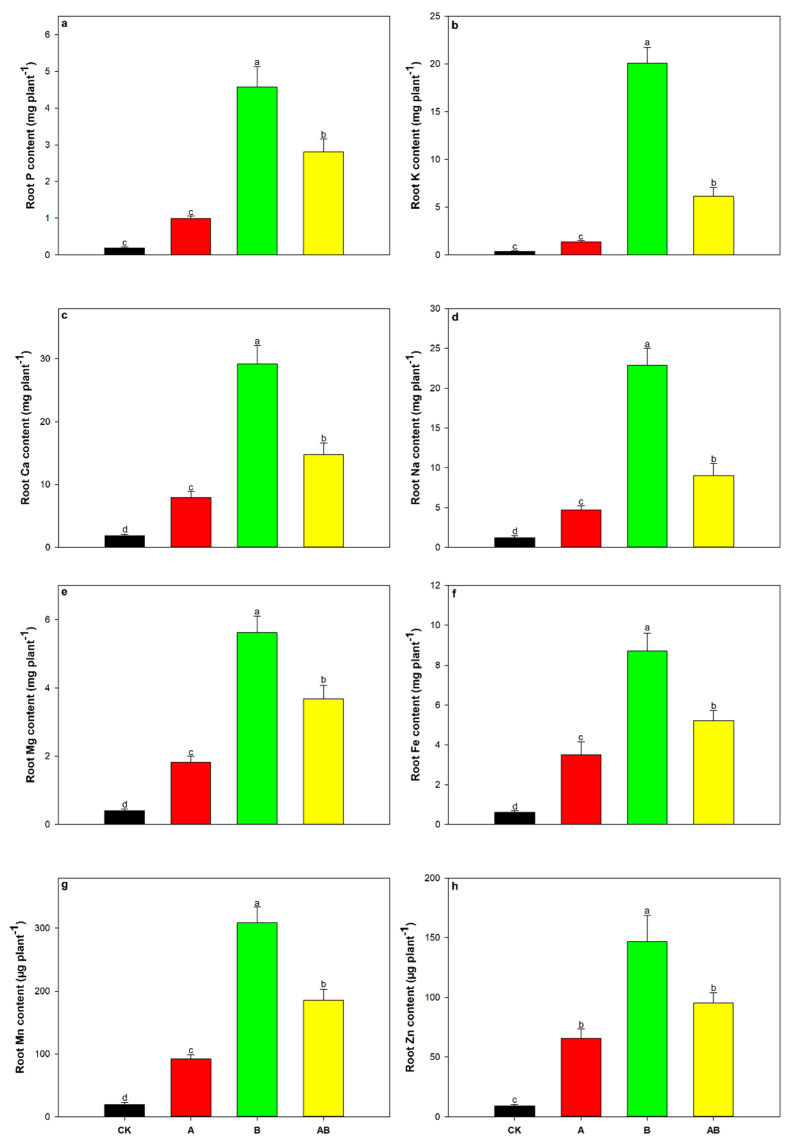
Effects of AMFs and biochar on root (**a**) P, (**b**) K, (**c**) Ca, (**d**) Na, (**e**) Mg, (**f**) Fe, (**g**) Mn, and (**h**) Zn contents. Data are means of five replicates ±SEs. Different letters above the columns indicate significant differences according to Duncan’s test at *p* < 0.05. CK, control; A, arbuscular mycorrhizal fungi; B, biochar; AB, arbuscular mycorrhizal fungi and biochar.

Maize shoot nutrients, including P, K, Ca, Na, Mg, Fe, Mn, and Zn, were significantly influenced by biochar and AMFs, as well as the interaction between biochar and AMFs (Table 2, Figure 4a–h). Compared with the control treatment, shoot P, K, Ca, Mg, Mn, and Zn contents were significantly increased by both the individual and dual applications of AMFs and biochar (Figure 4a–c,e,g,h). The dual application of AMFs and biochar significantly increased shoot Na content by 54.2% (Figure 4d), and the shoot Fe content was significantly increased by a factor of 1.8 by biochar addition compared with the control (Figure 4f). Compared with the control treatment, all shoot nutrient contents were substantially increased by the individual or dual application of AMFs and biochar, although root P and K contents were not significantly affected by AMFs (Figure 5a–h). For all root nutrients, the highest value was found in the biochar treatment group, followed by the dual AMF and biochar treatment, AMF-only treatment, and the control treatment groups (Figure 5a–h).

## 4. Discussion

### 4.1. Effects of AMFs

Plant nutrients play a critical role in driving a myriad of fundamental ecological processes, such as photosynthesis, plant growth and competition, pathogen infection, decomposition, and coupled biogeochemical cycling [25,26]. The extraradical mycelium of AMFs can expand the root surface, facilitating the acquisition of nutrients from soil [27,28]. There was a decrease in soil nutrient concentrations (e.g., NH_4_^+^-N, NO_3_^−^-N, and A-P) in the AMF treatment group, which may have been due to the high nutrient uptake promoted by AMFs in both roots and shoots, as shown in this study (particularly P) and earlier ones [29,30,31]. Furthermore, AMFs influence soil phosphatase enzymatic activity and soil physicochemical properties by improving phosphorus availability to the host plant [16]. Alkaline phosphatase activity was significantly higher in mycorrhizal roots than in non-mycorrhizal maize roots. Similarly, Liu et al. [32] detected mycorrhiza-specific phosphatase (MPSase) only in the root extract colonized by mycorrhizal fungi compared with the control treatment. 

Photosynthetic pigments, such as chlorophyll and carotenoid pigments, are essential for capturing light energy during photosynthesis [33]. The mycorrhiza treatment resulted in a significant increase in chlorophyll a, chlorophyll b, and total chlorophyll contents, reflecting considerable enhancements in the photosynthetic capacity and efficiency of maize [34]. Our results agree with those obtained by Malik et al. [35], who found that photosynthetic pigments increased in the presence of AMFs. The release of hormonal signals that promote chloroplast formation may potentially play a role, as well as the nutritional intake of elements including phosphorus and magnesium [36].

### 4.2. Effects of Biochar

Biochar application resulted in increased maize nutrient uptake and growth, which was consistent with findings of previous studies [37,38]. This may be because biochar can act as a nutrient source (e.g., N, P, and K) to directly improve plant growth [39]. In this study, we found that applying biochar to soil significantly enhanced the amount of accessible phosphorus in the soil. Furthermore, biochar can improve the physical and chemical properties of soil, such as bulk density, porosity, and water storage capacity, thus increasing the retention of water and nutrients and decreasing nutrient leaching [40]. In addition, biochar usage greatly increased soil alkaline phosphatase activity, which may have been a reaction to the recycling of organic phosphorus in soil. Biochar application can boost photosynthesis and chlorophyll content in a variety of plants [41,42]. However, neither the chlorophyll nor carotenoid content was influenced by biochar application in our experiment. Similarly, Rehman et al. [43] failed to find any relationship between the use of biochar and chlorophyll content in wheat and rice. The effect of biochar on photosynthetic pigments may depend on biochar type, application rate, soil conditions, and plant species [44].

### 4.3. Combined Effect of AMFs and Biochar

Biochar-induced increases in the mycorrhizal colonization rate have been observed in previous studies [45,46], as a result of changes in soil properties, alterations in soil microbial activities, or provision of refugia for AMFs [18,47]. Consequently, the combined application of biochar and AMFs may have a synergistic effect on plant growth [48]. However, biochar application significantly diminished the mycorrhizal colonization of maize in this study. Moreover, although the dual application of AMFs and biochar increased maize performance compared with the control, the benefits of dual application never exceeded the unique effect of biochar application alone. Our results are inconsistent with a previous study by Jabborova et al. [49], who found that the combined application of biochar and AMFs had a greater impact on *Spinacia oleracea* growth, root morphological traits, physiological properties, and soil enzymatic activities than individual application. The results did not support our hypothesis that the dual treatment has synergistic effects on plant performance. 

Previous studies have shown that the release of biochar P into soil can inhibit AM colonization and external hyphal growth [50], consequently diminishing the host’s nutrient uptake and benefits from AMFs. In this experiment, there was a profound increase in soil available P following biochar application. Furthermore, mycorrhizal root colonization was significantly negatively correlated with soil available phosphorus (Figure S1). The mycorrhizal dependency was 80.57% in non-biochar amended soil, but it decreased to −28.67% after biochar was added to the soil. The negative value of mycorrhizal dependency indicated a parasitic association between mycorrhizal fungi and maize plants when biochar was applied to soil [51,52]. In the present study, we show for the first time that the colonization and benefits of AMFs in maize can be reduced by the presence of biochar. Similarly, LeCroy et al. [53] reported that, under high N conditions, the combined biochar and AMF treatment decreased plant biomass compared with the treatment without biochar. In addition, previous studies showed that the combined application of biochar and AMF showed the significant effect of improving maize growth, especially under nutrient-limited, saline, or water stress [54,55,56]. The combined effect of biochar and AMF on plant growth may vary for different biochar types (i.e., biochar feedstock and production temperature) and application rates [57,58]. For example, biochar produced from leaves improved soil P availability more than that produced from woodchip biochar [59], and biochar produced at a higher temperature increased AMF root colonization and spore germination more than that produced at a lower temperature, due to polycyclic aromatic hydrocarbons (PAHs) produced by biochar at low temperatures [46]. Besides that, wide variability in morphological, physiological, molecular, and biochemical responses to biochar and AMFs have been observed among different maize varieties [60,61,62]. Since only one maize variety (Zhengdan 958) was used in our present experiment, inconsistent results may be obtained with other varieties [54]. The use of AMFs that are compatible with biochar and crop varieties combinations may provide satisfactory results [63]. For example, biochar combined with *Claroideoglomus etunicatum* showed a more profuse root system in strawberries than biochar combined with *C. pellucida* or mycorrhizal communities [64]. Our results suggest that applying biochar to soil may reduce AMF development and, consequently, mycorrhizal benefits [65,66,67].

## 5. Conclusions

Individual application of AMFs and biochar had positive effects on maize shoot, root, and total biomass; plant height; basal diameter; leaf number; the majority of mineral content in shoots and roots (P, K, Ca, Na, Mg, Fe, Zn, and Mn); and alkaline phosphatase activity. In addition, the chlorophyll a, chlorophyll b, and total chlorophyll contents in maize leaves were significantly increased by AMF inoculation. However, the combination of biochar and AMFs had no synergistic effects on maize performance in the present study. Furthermore, we show for the first time that biochar addition to soil significantly reduces mycorrhizal colonization in maize roots, thus reducing mycorrhizal benefits. Our study indicates a parasitic association between mycorrhizal fungi and maize plants when biochar is applied to soil. We conclude that biochar and AMF application can affect maize growth, nutrient uptake, physiological properties, and soil enzymatic activity. Our study can provide vital information for further resource use optimization in agroecosystems.

## Figures and Tables

**Table 1:** Mycorrhizal colonization rates of maize following different treatments.

Treatment	Mycorrhizal Colonization Rate
A	52.77 ± 4.74 a
AB	31.52 ± 2.47 b

Data represent the means of five replicates ±SEs. Values followed by different letters were significantly different according to Duncan’s test at *p* < 0.05. A, arbuscular mycorrhizal fungi; AB, arbuscular mycorrhizal fungi and biochar.

**Table 2:** Probabilities of significance calculated using a two-way ANOVA for the main treatment effects and treatment interactions of the measured variables.

Variable Measured	A		B		A × B	
	*F*	*P*	*F*	*P*	*F*	*P*
pH	0.14	0.71	0.76	0.40	0.69	0.42
NH_4_^+^-N	2.58	0.13	12.40	**	18.11	***
NO_3_^−^-N	159.78	***	200.84	***	135.80	***
A-P	6.24	*	108.27	***	5.47	*
DOC	8.48	*	5.08	*	0.54	0.47
MBC	19.66	***	22.15	***	35.43	***
MBN	0.22	0.65	11.31	**	1.55	0.23
Alkaline phosphatase	0.13	0.72	1.07	0.32	5.00	*
Shoot biomass	32.80	*	205.00	***	84.86	***
Root biomass	3.33	0.09	194.54	***	52.22	***
Total biomass	23.66	***	216.35	***	81.37	***
Root–shoot ratio	3.72	0.07	58.41	***	6.25	*
Chlorophyll a	0.97	0.34	0.46	0.51	8.70	**
Chlorophyll b	0.45	0.51	2.64	0.12	4.83	*
Total chlorophyll	0.58	0.46	3.08	0.10	8.51	**
Carotenoid	0.88	0.36	9.47	**	5.80	*
Shoot P content	11.26	**	82.08	***	22.29	***
Shoot K content	23.14	***	343.79	***	37.92	***
Shoot Ca content	13.18	*	35.70	***	34.25	***
Shoot Na content	10.14	**	12.77	**	5.51	*
Shoot Mg content	49.56	***	25.01	***	96.83	***
Shoot Fe content	9.07	**	25.71	***	8.69	**
Shoot Mn content	16.84	***	193.23	***	16.84	***
Shoot Zn content	22.26	***	39.69	***	54.48	***
Root P content	2.12	0.17	85.91	***	14.89	**
Root K content	48.00	***	170.26	***	63.18	***
Root Ca content	5.32	*	89.56	***	32.50	***
Root Na content	3.46	0.08	137.64	***	56.45	***
Root Mg content	2.59	0.13	187.01	***	48.65	***
Root Fe content	2.34	0.15	96.60	***	42.60	***
Root Mn content	1.28	0.28	242.22	***	69.45	***
Root Zn content	0.05	0.83	45.92	***	19.15	***

A, arbuscular mycorrhizal fungi; B, biochar; A × B, AMFs and biochar interaction; A-P, available phosphorus; DOC, dissolved organic carbon; MBC, microbial biomass carbon; MBN, microbial biomass nitrogen. * *p* < 0.05; ** *p* < 0.01; *** *p* < 0.001.

**Table 3:** Effects of arbuscular mycorrhizal fungi and biochar on soil properties and alkaline phosphatase activity.

Treatment	pH	NH_4_^+^-N (mg kg^−1^)	NO_3_^−^-N (mg kg^−1^)	A-P (mg kg^−1^)	DOC (mg kg^−1^)	MBC (mg kg^−1^)	MBN (mg kg^−1^)	Alkaline Phosphatase (nmol·g^−1^·d^−1^)
CK	8.39 ± 0.06 a	5.51 ± 0.49 a	23.93 ± 1.3 a	7.12 ± 0.69 c	71.41 ± 6.84 b	299.01 ± 18.18 a	18.6 ± 1.76 a	341.23 ± 59.56 b
A	8.49 ± 0.04 a	3.59 ± 0.35 b	4.48 ± 0.75 b	6.14 ± 0.24 c	86.44 ± 9.04 ab	147.43 ± 12.9 c	17.56 ± 1.09 a	2022.99 ± 253.72 a
B	8.37 ± 0.18 a	2.97 ± 0.04 b	3.25 ± 0.37 b	85.88 ± 12.21 a	81.9 ± 3.29 b	143.45 ± 11.88 c	12.4 ± 1.42 b	2052.78 ± 792.99 a
AB	8.32 ± 0.05 a	3.83 ± 0.26 b	2.46 ± 0.41 b	55.99 ± 1.74 b	107.06 ± 7.16 a	165.61 ± 14.63 c	14.7 ± 0.96 ab	1162.71 ± 310.19 ab

Data are means of five replicates ±SEs. Values within a column followed by different letters were significantly different according to Duncan’s test at *p* < 0.05. CK, control; A, arbuscular mycorrhizal fungi; B, biochar; AB, arbuscular mycorrhizal fungi and biochar; A-P, available phosphorus; DOC, dissolved organic carbon; MBC, microbial biomass carbon; MBN, microbial biomass nitrogen.

**Table 4:** Mycorrhizal dependency of inoculated maize following different biochar treatments.

Treatment	Mycorrhizal Dependency
Without biochar	80.57 ± 1.63a
With biochar	−28.67 ± 3.93b

Data represent the means of five replicates ±SEs. Values followed by different letters were significantly different according to Duncan’s test at *p* < 0.05.

**Table 5:** Probabilities of significance calculated using a three-way ANOVA for the main treatment effects and treatment interactions of maize height, basal diameter, and leaf number.

Variable Measured	A		B		T		A × B		A × T		B × T		A × B × T	
	*F*	*P*	*F*	*P*	*F*	*P*	*F*	*P*	*F*	*P*	*F*	*P*	*F*	*P*
Maize height	135.17	***	330.47	***	761.03	***	305.11	***	32.73	***	42.21	***	38.15	***
Basal diameter	202.22	***	135.62	***	300.61	***	90.99	***	49.11	***	33.32	***	19.12	***
Leaf number	180.04	***	170.04	***	133.18	***	72.32	***	16.99	***	12.70	***	20.70	***

A, arbuscular mycorrhizal fungi; B, biochar; T, sampling time; A × B, AMFs and biochar interaction; A × T, AMFs and sampling time interaction; B × T, biochar and sampling time interaction; A × B × T, AMF, biochar and sampling time interaction. *** *p* < 0.001.

## Data Availability

Not applicable.

## Supplementary Materials

The following supporting information can be downloaded at: https://www.mdpi.com/article/10.3390/jof8121275/s1, Figure S1: Pearson correlation analysis (r) between maize growth parameters and soil properties at harvest, Figure S2: Effects of AMFs and biochar on shoot (a) P, (b) K, (c) Ca, (d) Na, (e) Mg, (f) Fe, (g) Mn, and (h) Zn concentrations, Figure S3: Effects of AMFs and biochar on root (a) P, (b) K, (c) Ca, (d) Na, (e) Mg, (f) Fe, (g) Mn, and (h) Zn concentrations.
