# Supplementary material for: Effects of Arbuscular Mycorrhizal Fungi and Biochar on Growth, Nutrient Absorption, and Physiological Properties of Maize (Zea mays L.)
Source: J Fungi (Basel). 2022 Dec 5;8(12):1275. doi: 10.3390/jof8121275 (PMC9782859; doi:10.3390/jof8121275)
Supplement: Supplementary file 1 [file jof-08-01275-s001.zip › jof-2033901-supplementary.pdf]

Journal of
Fungi

MDPI

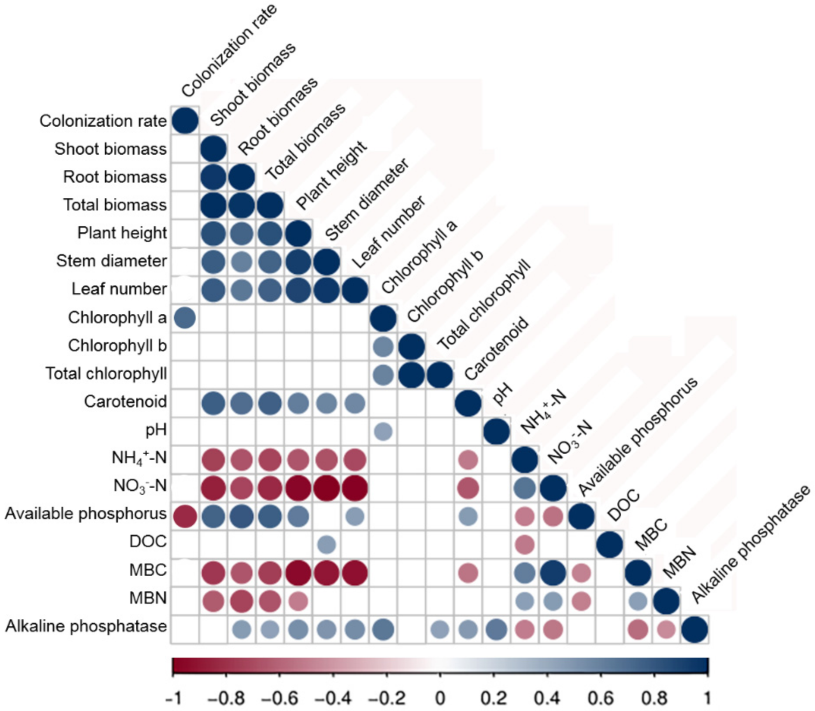

Figure S1. Pearson correlation analysis (*r*) between maize growth parameters and soil properties at harvest. Positive and negative correlations are displayed in blue and red, respectively. Color intensity and the size of the circles are proportional to the correlation coefficients. Null when the correlation is not significant ($P \geq 0.05$).

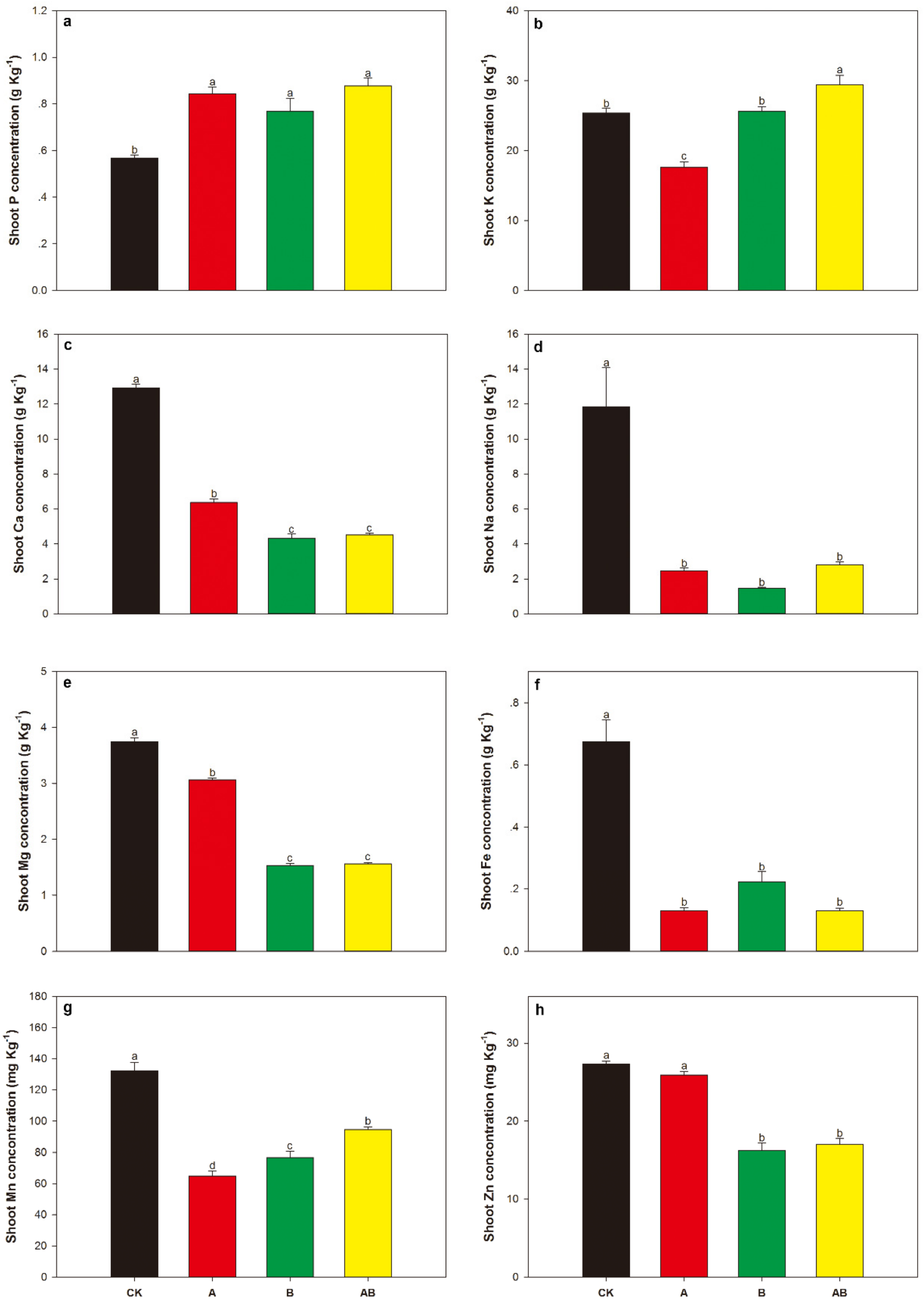

Figure S2. Effects of AMF and biochar on shoot **(a)** P, **(b)** K, **(c)** Ca, **(d)** Na, **(e)** Mg, **(f)** Fe, **(g)** Mn, and **(h)** Zn concentrations. Data are means of five replicates ± SE. Different letters above the columns indicate significant differences according to Duncan's test at $P < 0.05$. CK, control; A, arbuscular mycorrhizal fungi; B, biochar; AB, arbuscular mycorrhizal fungi and biochar.

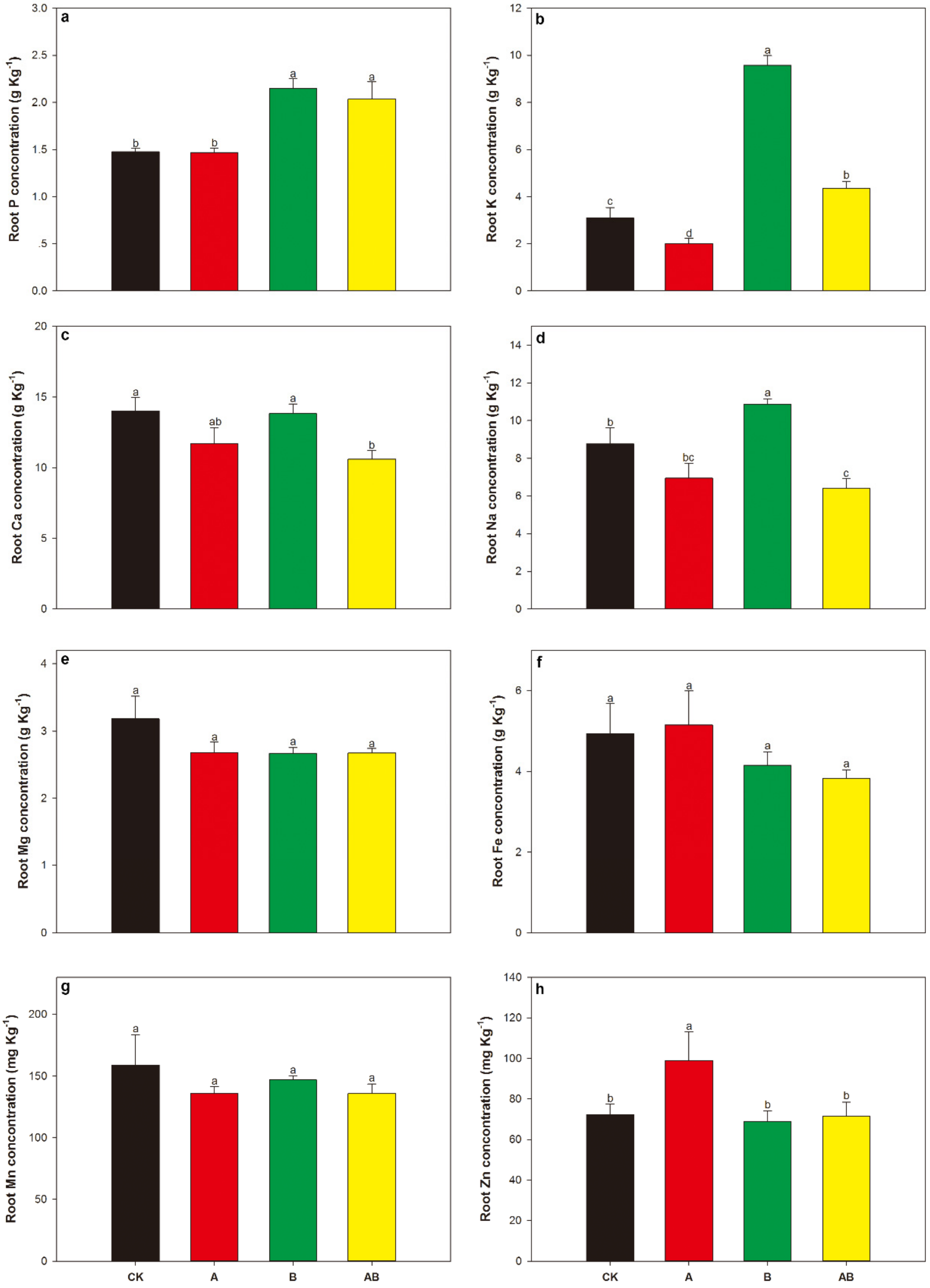

Figure S3. Effects of AMF and biochar on root **(a)** P, **(b)** K, **(c)** Ca, **(d)** Na, **(e)** Mg, **(f)** Fe, **(g)** Mn, and **(h)** Zn concentrations. Data are means of five replicates ± SE. Different letters above the columns indicate significant differences according to Duncan's test at $P < 0.05$. CK, control; A, arbuscular mycorrhizal fungi; B, biochar; AB, arbuscular mycorrhizal fungi and biochar.
